# A model decomposition method for the real-time non-line-of-sight imaging

**DOI:** 10.1016/j.isci.2026.115828

**Published:** 2026-04-21

**Authors:** Peng Yang, Zewei Wang, Yinghui Guo, Xiaoying Li, Mingbo Pu, Hengshuo Guo, Mingfeng Xu, Fei Zhang, Yuanmao Wang, Xiangang Luo

**Affiliations:** 1 State Key Laboratory of Optical Field Manipulation Science and Technology, Institute of Optics and Electronics, Chinese Academy of Sciences, Chengdu 610209, China; 2 College of Materials Science and Opto-Electronic Technology, University of Chinese Academy of Sciences, Beijing 100049, China; 3 Research Center on Vector Optical Fields, Institute of Optics and Electronics, Chinese Academy of Sciences, Chengdu 610209, China; 4 Sichuan Provincial Engineering Research Center of Digital Materials, Chengdu 610290, China; 5 Tianfu Xinglong Lake Laboratory, Chengdu 610290, China

**Keywords:** Applied sciences, Engineering

## Abstract

Real-time non-line-of-sight (NLOS) imaging faces a fundamental trade-off between acquisition efficiency and reconstruction quality. Although transient methods achieve high visual fidelity, they depend on extensive data collection. Regularization-based approaches allow undersampled reconstruction but often incur prohibitive computational costs. To overcome these limitations, we introduce MD-NLOS, a model decomposition method that formulates NLOS reconstruction as a least absolute shrinkage and selection operator (LASSO) problem improved by spectral filtering. By solving the optimization in the frequency domain, the method achieves notable computational efficiency. Furthermore, the reformulated problem can be effectively solved using simple gradient descent, avoiding the need for complex optimization schemes. The results show that our method reconstructs synthetic 256 × 256 and experimental 128 × 128 images, using only 36 and 64 sampling points, with reconstruction times of 3.1 and 4.6 s, respectively, yielding a structural similarity index (SSIM) of 0.7352, which is approximately 6-fold higher than that of FK.

## Highlights

• MD-NLOS enables fast NLOS imaging from sparse transient measurements
• NLOS reconstruction is reformulated as a non-negative LASSO problem
• Spectral filtering and GPU-parallelizable optimization improve efficiency
• 64 samples recover 128 × 128 images in 4.6 s

## Graphical abstract

**Figure:**
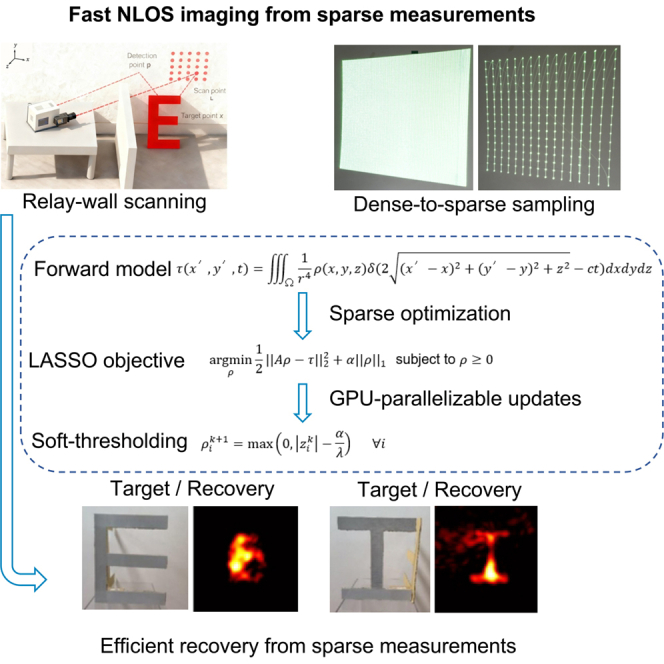

## Introduction

Non-line-of-sight (NLOS) imaging is an emerging computational imaging technique that has rapidly developed in recent years, aimed at reconstructing hidden objects outside the conventional line of sight.^1^^,^^2^^,^^3^^,^^4^^,^^5^^,^^6^ This technology holds potential applications in security surveillance, search and rescue, and autonomous driving. To make NLOS imaging practical, achieving real-time performance—that is, rapid image reconstruction and minimal acquisition time—is essential.

A variety of NLOS imaging methods have been developed, including acoustic imaging,^7^^,^^8^ transient imaging,^9^^,^^10^^,^^11^ thermal imaging,^12^ speckle correlation,^13^^,^^14^^,^^15^ wavefront shaping,^16^^,^^17^^,^^18^ occlusion-based imaging,^19^^,^^20^^,^^21^ and so forth. Recently, NLOS studies have increasingly exploited multidimensional light-field cues, particularly polarization and spectral diversity, to mitigate the ill-posedness of reconstruction in passive or low-signal-to-noise ratio (SNR) scenarios. Polarimetric NLOS has demonstrated improved recoverability by incorporating degree of linear polarization (DoLP) information through polarization encoding and learning-based inversion, leading to more robust reconstructions than intensity-only baselines.^22^ Complementarily, hyperspectral and multispectral NLOS leverage joint spatial-spectral correlations and cross-band complementarity (Visible/Short-Wave Infrared/Long-Wave Infrared band) to enhance structural and color fidelity, albeit with added demands on acquisition, calibration, and computation.^23^^,^^24^

Among these, transient techniques acquire information in the form of time-of-flight (TOF) histograms—recording photon intensity and arrival time—and have evolved into the most prominent research area. Polarization-aware time-gated NLOS^25^ incorporates polarization cues to alleviate missing-cone effects and improve geometric recovery. In parallel, infrared-active NLOS^26^ using single-photon detectors extends time-resolved NLOS to longer wavelengths and improves performance under low-SNR conditions via physics-informed reconstruction pipelines. Single-shot full-Stokes polarimetric NLOS^27^ demonstrates that polarization encoding combined with lightweight learning-based inversion can reduce acquisition burden, albeit with increased sensitivity to calibration and data-domain generalization. What’s more, several typical methods, such as frequency-wavenumber migration (FK),^28^ the light-cone transform (LCT),^29^ and filtered back-projection (FBP),^30^ have been developed to invert TOF histograms and reconstruct hidden scenes efficiently. These analytical reconstruction algorithms offer computational simplicity and are compatible with real-time processing. Nevertheless, their reliance on point-by-point mechanical scanning of the relay wall results in long data acquisition times, severely limiting real-time performance. Although undersampling can be employed to enhance imaging speed, it exacerbates the large-scale ill-posed equation problems, thus causing these methods to exhibit limited robustness under low sampling rates. To enhance image quality, the scanning field-of-view is expanded via liquid crystals, and the resultant resolution is compensated for using correlation-based computational algorithms.^31^ Additionally, the extraction of polarization information through vectorial optical field manipulation presents a viable strategy for improving performance under low-SNR conditions,^32^ and using binocular meta-lens also works.^33^

Iterative optimization methods^34^^,^^35^^,^^36^^,^^37^^,^^38^^,^^39^^,^^40^^,^^41^ that incorporate prior knowledge or regularization can improve reconstruction robustness under undersampling but often at the cost of heavy computation. Although deep learning methods^42^^,^^43^^,^^44^^,^^45^ have been introduced to balance acquisition time and computational efficiency, their practicality is often limited by the substantial demand for training data and constrained generalization capability. Alongside backend algorithms for real-time processing, methods such as dimensionality-reducing circular scanning^38^ and the single-photon avalanche diode (SPAD) array^46^^,^^47^^,^^48^^,^^49^ deployment are also employed. However, SPAD arrays suffer from crosstalk, which impairs temporal and resultant spatial resolution, in addition to incurring higher system costs. Circular scanning, meanwhile, is limited by the geometrical constraints of its scan pattern on the relay wall. Therefore, balancing reconstruction quality, computational efficiency, and acquisition time becomes the central challenge of NLOS imaging.

In this paper, a computationally efficient LCT-based model decomposition method is introduced to solve the ill-posed problem (MD-NLOS). We reformulate the confocal transient model into a decomposition between a known system, point spread function (PSF), and the unknown albedo and further derive a frequency-domain point-wise multiplication form, which significantly reduces computational complexity and provides an efficient way to compute gradients—forming the basis for real-time reconstruction. We show that, in this problem, the system matrix columns are highly coupled and non-differentiable at zero, which make it difficult for the conventional gradient descent method to determine convergence, and may oscillate. Additionally, we add a quadratic term to the objective function and an explanation for its natural source and decoupling effect, allowing updates to be stably executed in a very simple gradient descent form. It restructures the operator so that the core computations become GPU friendly. Besides, in the NLOS scenario, typical hidden targets have their albedo occupying only a finite space support on the 3D-reconstructed grid, resulting in a significant sparse structure in the spatial domain. Model decomposition shows that transient measurements can be decomposed into two parts: unknown scene albedo distribution and unrelated system response. Therefore, the iterative solving process is the process of sparse recovery. Thus, an *L*_1_ term is added as a penalty function to promote sparsity. Due to non-ideal effects such as SPAD dead time, strict Poisson modeling not only brings limited benefits but also significantly increases the complexity of gradient calculation. Therefore, Gaussian assumption is adopted to simplify the calculation. In addition, the albedo is non-negative, and we express the problem reconstruction as a least absolute shrinkage and selection operator (LASSO) form with non-negative constraints. Finally, the introduction of spectral filtering improves our method’s performance of noise resistance and robustness, facilitating quality reconstructions. On synthetic data, the MD-NLOS algorithm accurately reconstructs object contours with 1,820-fold sampling reduction while maintaining visual fidelity. Moreover, when the number of sampling points is reduced from 256 to 36, the structural similarity index (SSIM) varies by less than 0.03. On experimental data, the method achieves 128 × 128 reconstructions with 64 sampling points in 4.6 s, demonstrating nearly two orders of magnitude faster computation compared with deconvolution optimization-based non-line-of-sight (DO-NLOS) imaging algorithm.^35^ For 64 × 64 datasets—both public synthetic and experimental—our method achieves imaging speeds comparable to those of the direct methods LCT, FBP, and FK, completing reconstruction within 1 s. The results confirm that MD-NLOS effectively mitigates the trade-off among image quality, speed, and sampling density, offering a promising route toward real-time NLOS imaging systems.

## Results

### Forward model based on LCT decomposition

Based on the physical model of confocal NLOS imaging using the LCT method, the measured transient histogram *τ*(*x*′,*y*′,*t*) is acquired by the SPAD. *ρ*(*x*,*y*,*z*) represents the unknown scene albedo in the 3D reconstruction domain Ω; the relationship between them can be written in Equation 1:

(Equation 1) $$\begin{array}{c} \tau \left(x^{\prime },y^{\prime },t\right)={∭}_{\Omega }\;\frac{1}{r^{4}}\rho \left(x,y,z\right)\delta \left(2\sqrt{{\left(x^{\prime }-x\right)}^{2}+{\left(y^{\prime }-y\right)}^{2}+z^{2}}-ct\right)dxdydz \end{array}$$

where *r* = *ct*/2, *δ*(·) is the Dirac delta function, and its geometric interpretation corresponds to a spherical constraint. The imaging principle is based on geometric constraints and presented in STAR Methods, imposing a 4D spatiotemporal hypocone constraint: a point *X*(*x*,*y*,*z*) on the hidden object contributes to the measurement at time *t* only if it satisfies ${\left(x^{\prime }-x\right)}^{2}+{\left(y^{\prime }-y\right)}^{2}+z^{2}={\left(ct/2\right)}^{2}$.

The core of model decomposition methods lies in model selection, and the key to selecting an appropriate model involves balancing model accuracy against inversion time: although some models offer higher accuracy, their long inversion times often render them impractical for real-time applications. The selection of NLOS models is discussed in STAR Methods.

**Figure 1:**
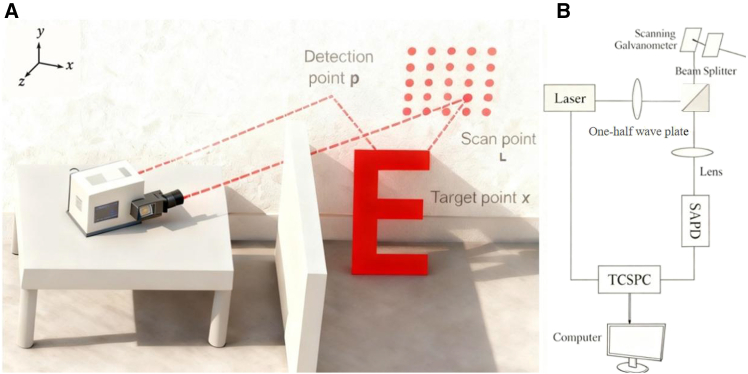
NLOS imaging principle and confocal optical path (A) Schematic of the time-of-flight-based NLOS imaging principle. (B) Schematic of the optical path used to realize confocal NLOS imaging.

Figure 1 illustrates a schematic of the entire imaging system. As shown in Figure 1A, in the triple-bounce NLOS configuration—where the laser undergoes three reflections (wall, object, and wall) from emission to detection—each scanning point *L*(*x*′,*y*′) on the wall yields a histogram *τ*(*x*′,*y*′,*t*) collected by the SPAD at the detector location *p*. This histogram is accumulated by a time-correlated single-photon counting (TCSPC) module over repeated cycles. When the scan point *L* coincides with the detection point *p*, a confocal condition is achieved. As shown in Figure 1B, a beam splitter overlaps the emitted and returning optical paths. The workflow is as follows: a pulsed laser emits light that is split into two paths by the beam splitter. One path is directed to a scanning galvanometer to illuminate the relay wall, while the other continues straight and is completely absorbed by an absorber (serving as a calibration signal to mark the start of the TOF).

To discretize the Equation 1 for numerical computation, variable substitutions *v*=(*ct*/2)^2^ and $z=\sqrt{u}$ are applied; the above model can be transformed into a 3D convolution form and expressed as a matrix equation:

(Equation 2) $$\begin{array}{c} R_{t}\tau =h*R_{z}\rho \end{array}$$

Here, *R*_*t*_ and *R*_*z*_ are resampling transformation matrices acting on the temporal and spatial dimensions, respectively. ∗ represents convolution. $\tau \in R_{+}^{n_{x}n_{y}n_{t}}$, $\rho \in R_{+}^{n_{x}n_{y}n_{z}}$, $h\in R_{+}^{n_{x}n_{y}n_{h}\times n_{x}n_{y}n_{h}}$, $R_{z}\in R_{+}^{n_{x}n_{y}n_{h}\times n_{x}n_{y}n_{t}}$, $R_{t}\in R_{+}^{n_{x}n_{y}n_{h}\times n_{x}n_{y}n_{z}}$.

Equation 2 can be further rewritten in the frequency domain as follows:

(Equation 3) $$\begin{array}{c} F\left\{R_{t}\tau \right\}=H⊙F\left\{R_{z}\rho \right\} \end{array}$$

Here, F is the 3D discrete Fourier transform. ⊙ denotes the point-wise multiplication. *H* represents the Fourier transform of the PSF. Equation 3 allows to significantly reduce the computational complexity. In particular, working in the Fourier domain enables efficient computation of the gradient ∇*g*(*ρ*^*k*^) in Equation 9, which lays the foundation for subsequent real-time reconstruction. For simplicity, however, we omit explicit use of this frequency-domain approach in subsequent derivations.

In essence, the measured transient histogram *τ*(*x*′,*y*′,*t*) can be decomposed into two components: the known system PSF and the unknown scene albedo distribution *ρ*(*x*,*y*,*z*).The PSF is determined by the system geometry and propagation characteristics of light, including known factors such as the scanning area dimensions and the speed of light *c*.

To simulate undersampling conditions, a binary sampling mask *M*(*x*′,*y*′,t) is introduced:

(Equation 4) $$\begin{array}{c} \tau \left(x^{\prime },y^{\prime },t\right)=M\left(x^{\prime },y^{\prime },t\right)⊙\tau_{\text{full}}\left(x^{\prime },y^{\prime },t\right) \end{array}$$

*M*(*i*,*j*,*t*) = 1 indicates that the corresponding point is sampled, while *M*(*i*,*j*,*t*) = 0 indicates missing data.

### LASSO framework for denoising and efficient computation

It is important to note that due to non-ideal effects like dead time^43^ in the SPAD detector, the actually measured time histogram data *τ* do not strictly follow Poisson’s distribution. Using Poisson’s noise model in reconstruction not only offers limited improvement in model accuracy but also significantly increases the complexity of gradient calculation, which is detrimental to real-time algorithm solving. We, therefore, assume that *τ* follows a Gaussian distribution to simplify the computation.

For the NLOS inversion problem defined above, we adopt a LASSO optimization framework to achieve a fast, sparse, and stable reconstruction solution. The optimization objective is formulated as follows:

(Equation 5) $$\begin{array}{c} \underset{\rho }{\arg\min}\;\frac{1}{2}{\left\|A\rho -\tau \right\|}_{2}^{2}+\alpha {\left\|\rho \right\|}_{1}\;\text{subject}\;\text{to}\;\rho \geq 0 \end{array}$$

where the first term represents the negative log likelihood under the Gaussian noise assumption; detailed derivation is shown in the STAR Methods. The second term promotes sparsity of the solution, and the non-negativity constraint ensures physical validity because light intensity cannot be negative, which is enforced via the projection operator *max*(a,0) in each iteration. The regularization weight *α* controls the degree of sparsity: a larger *α* drives more components toward zero, effectively denoising the reconstruction by selecting only the most significant reflectance features. Mathematical meaning is shown in STAR Methods. In practice, we choose *α* proportional to the square root of the total photon count, following the guideline.^50^

When the gradient descent method was applied to solve Equation 5, it is noted that matrix *A* in this problem is neither orthogonal nor square, as the NLOS forward model is typically underdetermined and ill posed. As a result, the usual approach of simplifying the gradient by interchanging *A*^*T*^ and *A*^−1^ does not apply here, leading to the following gradient expression:

(Equation 6) $$\begin{array}{c} \nabla f\left(\rho \right)=A^{T}\left(A\rho -\tau \right)+\alpha \partial {\left\|\rho \right\|}_{1} \end{array}$$

where $f\left(\rho \right)=\frac{1}{2}{\left\|A\rho -\tau \right\|}_{2}^{2}+\alpha {\left\|\rho \right\|}_{1}$, ∂ is the subgradient operator.

In our problem, the columns of *A* are highly coupled, leading to high computational complexity and difficulty in solving *ρ*. Moreover, due to the non-differentiability of the *L*_1_ term at zero, even if *A* were orthonormal, one could not obtain a closed-form update for each *ρ*_*i*_ via simple soft-thresholding. The L_1_ term yields a continuum of solutions when the subgradient condition is zero, meaning that a conventional gradient descent can struggle to identify convergence and may oscillate in the flat regions of the objective.

To address these challenges, we augment the objective with a quadratic term $\lambda /2{\left\|\rho -\rho^{k}\right\|}_{2}^{2}$. This yields the modified problem:

(Equation 7) $$\begin{array}{c} \underset{\rho }{\arg\min}\;\frac{1}{2}{\left\|A\rho -\tau \right\|}_{2}^{2}+\alpha {\left\|\rho \right\|}_{1}+\frac{\lambda }{2}{\left\|\rho -\rho^{k}\right\|}_{2}^{2},s.t.\;\rho \geq 0 \end{array}$$

which leads to the following iterative update rule for the solution:

(Equation 8) $$\begin{array}{c} \rho^{k+1}=\rho^{k}-\frac{1}{\lambda }\nabla f\left(\rho^{k+1}\right) \end{array}$$

where the gradient is evaluated at the new iterate *ρ*^*k*+1^. The inclusion of the quadratic term resolves the issues mentioned above. Essentially, this term allows the algorithm to automatically detect when the optimum is reached during the update of *ρ*^*k*+1^. Although Equation 8 appears to assume prior knowledge of the optimal *ρ*^*k*+1^ (which seems contradictory), it, in essence, remains equivalent to a gradient descent step. Moreover, the quadratic term facilitates a “complete-the-square” decoupling of the optimization (see STAR Methods for details). Accordingly, we can rewrite the update in a more concise form as follows:

(Equation 9) $$\begin{array}{c} \rho^{k+1}=\underset{\rho }{\text{ar}\text{gmin}}\;\left\{\alpha {\left\|\rho \right\|}_{1}+\lambda {\left\|\rho -z^{k}\right\|}_{2}^{2}\right\},s.t.\;\rho \geq 0 \end{array}$$

where $z^{k}=\rho^{k}-\frac{1}{\lambda }\nabla g\left(\rho^{k}\right)$, and $g\left(\rho \right)=\frac{1}{2}\|A\rho -\tau {\|}_{2}^{2}$. Solving this subproblem (with the non-negativity constraint) yields a closed-form solution via soft-thresholding:

(Equation 10) $$\begin{array}{c} \rho_{i}^{k+1}=\max\left(0,\left|z_{i}^{k}\right|-\frac{\alpha }{\lambda }\right)\;\forall i \end{array}$$

We set the parameter *λ* adaptively at each iteration, using the Rayleigh quotient as the step size:

(Equation 11) $$\begin{array}{c} \lambda_{k}=\|A\left(\rho^{k}-\rho^{k-1}\right)\left|{|}_{2}^{2}/\|\rho^{k}-\rho^{k-1})\right|{|}_{2}^{2} \end{array}$$

The introduction of this proximal term decouples the linear dependence in *A* and addresses the non-smoothness of the *L*_1_ penalty, enabling stable and automatic convergence of the iterative updates. In fact, the overall algorithm can be interpreted as a forward-backward splitting process: the forward step computes a gradient update according to the *L*_2_ term, and the backward step applies a soft-thresholding operation to enforce the *L*_1_ sparsity penalty.

### Spectral filtering for the further improvement of imaging quality

Current method that exists perform spectral filtering on *τ* in the frequency domain to obtain higher-resolution images.^33^ However, simply imposing smoothing or bandpass “priors” on *τ* or *ρ* carries the risk of suppressing edges due to altered frequency spectra. To further improve noise robustness and suppress artifacts under severe undersampling, a spectral filtering step is introduced. Specifically, during the “selection” step of our LASSO-based algorithm—we consider the gradient ∇*g*(*ρ*^*k*^) = *A*^*T*^(*Aρ*^*k*^-*τ*). It is possible that the inner product value is less than the penalty coefficient *α*, causing the corresponding column of *A* that should be retained to be discarded, which means the loss of effective signal in *ρ*. Therefore, filtering the spectrum of the residual *Aρ*^*k*^-*τ* is a better choice that mitigates this risk before *Aρ*^*k*^-*τ* is mapped into *ρ* via *A*^*T*^, thereby influencing its values. The residual is transformed into the Fourier domain, bandpass filtered to remove low-frequency background fluctuations and high-frequency noise from weak NLOS signals, and then inverse transformed back into the temporal domain. This residual-domain filtering strategy improves the SNR without increasing the risk of distorting structural details.

### Experimental setup

To validate the proposed approach experimentally, a confocal NLOS imaging system was constructed using a 532 nm pulsed laser, with a pulse width of approximately 20 ps, the repetition rate of 12 MHz, and the average power of 50 mW. The timing jitter is approximately 80 ps, and the temporal resolution is 4 ps. The model of the SPAD is PDM, with a response range of 900–1,700 nm, a gate rise time of 2 ns, and a gate width of 1 ns–1.5 ms. The letters “I” and “E” were positioned at one corner of the scene. The letter “I” was oriented toward the relay plane at a distance of 0.5 m, while the letter “E” was oriented toward the relay plane at a distance of 1 m. A galvanometric scanner was then employed to uniformly scan a 0.8 m × 0.8 m area of a rough, white wall. With an acquisition time interval of 0.1 s, two distinct data were acquired: one for the letter “E” and one for the letter “I”, each with physical dimensions of 30 × 40 cm^2^. Each resulting transient data had dimensions of 128 × 128 × 4,096 (spatial × spatial × temporal). During subsequent processing, to streamline computation and handling, the data dimensions were downsampled to 128 × 128 × 512. In post-proceeding, all computations were performed on an NVIDIA RTX 4060 GPU.

### Results on our experimental data

**Figure 2:**
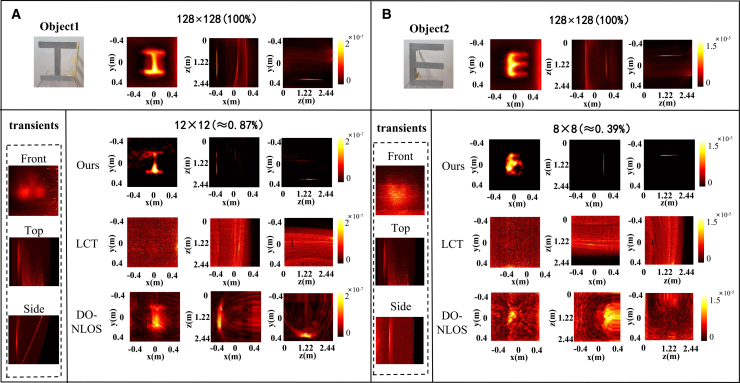
Reconstruction and runtime on experimental NLOS data The proposed method is compared with the direct method LCT and the iterative method DO-NLOS for different experimental targets, object 1 and object 2, at sampling rates of 0.87% and 0.39%, respectively. (A) The letter “I.” (B) The letter “E.”

For reference, we first applied the standard LCT algorithm to the fully sampled experimental data to obtain baseline reconstructions. Figures 2A and 2B show the three-view visualizations of the reconstructed hidden scenes using our MD-NLOS method at undersampling ratios of 0.87% (64 points) and 0.39% (36 points), respectively. The transient information collected by the detector is located in the lower left corner (as for TOF of the single point, see Figure S1). MD-NLOS could successfully recover both “I” and “E.” Compared with the fully sampled reference result, certain geometric distortions are reduced in our reconstructions—particularly for the second object (“E”). However, thanks to its strong noise suppression capability, our undersampled reconstruction actually provides more precise localization of the objects in three dimensions. As seen in the fully sampled image, the specific positions of objects cannot be accurately determined due to the interference of artifacts. For the more complex imaging scenario (multiple reflections), the performance of the proposed method is shown in Figure S2*.*

**Figure 3:**
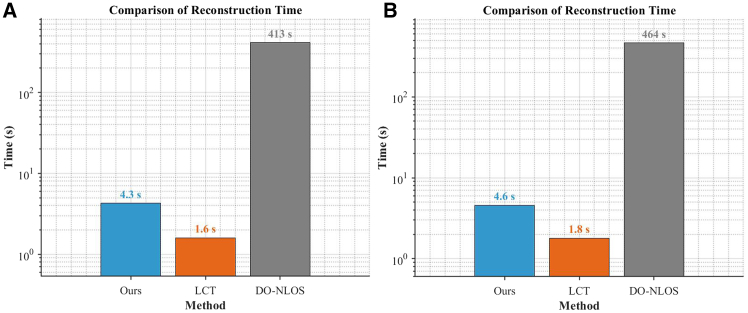
Logarithmic plot of reconstruction time for different methods (A) The target is the letter “I.” (B) The target is the letter “E.”

Figures 3A and 3B plot the reconstruction time (on a logarithmic scale) for different methods corresponding to the results in Figures 2A and 2B. Although each method’s runtime differs between the two target scenes, the overall trends are similar. Our method takes approximately three times longer than direct LCT, but it is about two orders of magnitude faster than the iterative DO-NLOS method, achieving a reconstruction speed that is close to real time.

### Results on our synthetic data

**Figure 4:**
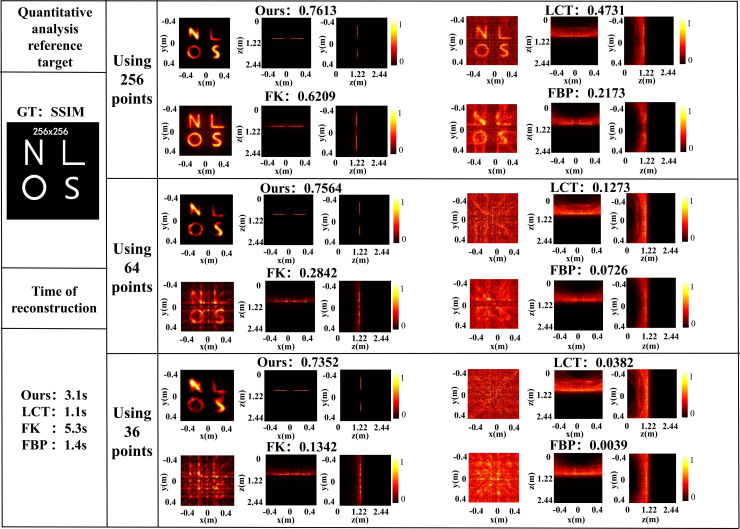
Reconstruction comparison on 256 × 256 synthetic NLOS data The SSIM-based comparison shows the reconstruction performance of different algorithms on 256 × 256 synthetic data under different undersampling conditions.

We further evaluated our method on synthetic NLOS datasets with various undersampling rates and compared the results against several representative analytical methods, including FBP, LCT, and FK. The simulation setup is consistent with the experimental configuration described above. We generated synthetic transient data following O’Toole et al.^29^ Figure 4 shows the ground-truth hidden target (a 256 × 256 pixels image) used for the simulation, which is assumed to lie 1 m in front of the relay wall.

We uniformly sampled 256, 64, and 36 points, corresponding to sampling ratios of 0.39%, 0.098%, and 0.055%, respectively. Figure 4 compares the reconstruction results obtained using different methods. For the data utilizing 256 sampling points, the FK method yielded the most complete outcome but introduced moderate noise concentrated in the central region. Although our method lost some edge information, it achieved the best noise suppression. FBP and direct LCT reconstructions are notably noisier, underscoring their weaker noise suppression ability than MD-NLOS, even in low-noise simulations. As the number of sampling points decreased further to 64 and 36, the reconstruction quality of all methods degraded to varying degrees. LCT and FBP were completely overwhelmed by noise, while FK exhibited artifacts. This demonstrates that under rather low-noise simulated conditions, compared with practical situation, our algorithm could effectively suppress the degradation in imaging quality caused by undersampling, successfully retaining basic information of the target. Our method achieved an SSIM of 0.7352 when using 36 samples, and the decrease in SSIM did not exceed 0.03 as the number of sample points decreased from 256 to 36. We also collected data on PSNR and RMSE metrics (see Table S1).

According to the scanning duration of 0.1 s per point, the total acquisition time of the proposed approach is only 3.6 s for 36 points, compared with nearly 2 h for fully scanning. This corresponds to 1820-fold reduction in acquisition time without sacrificing key visual information.

In this paper, compared with different sampling points, the data size dimension is the factor that determines the reconstruction time. Therefore, we only recorded the time consumption information of different methods, with a sampling point of 36. As can be seen from Figure 4, our method had a recovery time of 3.1 s, which is better than FK’s 5.3 s but inferior to LCT’s 1.1 s and FBP’s 1.4 s recovery time.

### Results on public synthetic and experimental data

To further assess the generalizability and robustness of our method, we evaluated MD-NLOS on publicly available datasets widely used in previous research.^28^^,^^29^ We selected one synthetic scene (“bunny” that contains complex, fine details, and hierarchical structure) and five experimental scenes: a pair of partially occluded letters “SU,” a discrete small object (“dot”), an outdoor scene (“S”), and the complex targets dragon and bike. For each scene, we used the LCT algorithm on fully sampled data to obtain a reference reconstruction.

**Figure 5:**
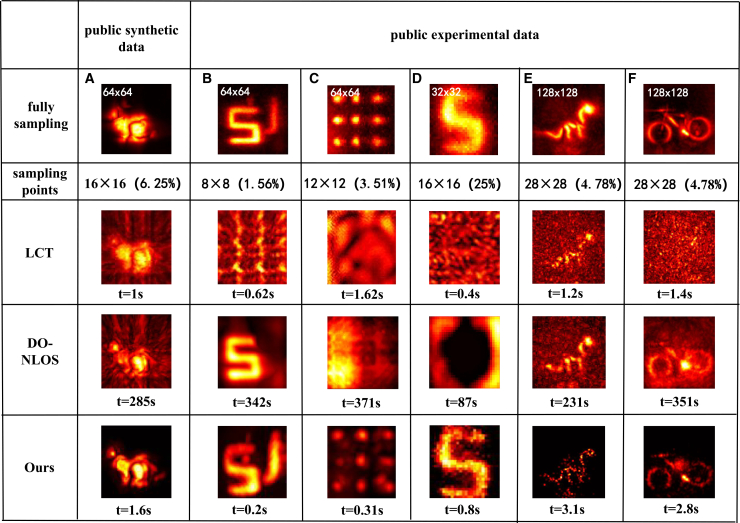
Comparison on public datasets (experimental and synthetic) and runtime (A) Composite “bunny.” (B) Continuous objects. (C) Discrete objects. (D) Outdoor data. The runtime of different methods is indicated below each image. (E) Dragon (30 min). (F) Bike (30 min).

Figure 5 compares the undersampled reconstruction results of different methods on these scenes. In the synthetic “bunny,” the LCT method recovers the object well with full data, but under sparse sampling, it could retain only a basic contour of the bunny. The DO-NLOS method recovers some detailed features in the bunny, but its advantage is largely masked by high noise. In contrast, our MD-NLOS method clearly stands out by preserving detailed structures, thanks to its strong denoising capability. For the “SU” letters scene (two letters on different planes, with the “U” partially occluded by the “S”), all methods, except LCT, primarily reconstructed the unobstructed “S.” Our method reconstructed the visible “S” clearly, and while it could not recover the occluded “U” as completely as the unoccluded letter, it successfully captured the U’s fundamental shape. On the other hand, for the “dot” (a very small discrete target) and the outdoor “S” scene, even our method failed to reconstruct a very clear image. We attribute this to the extremely low SNR in these scenarios: the “dot” target is very small, and the outdoor “S” scene is dominated by strong ambient noise. Finally, we achieved the same effect on complex experimental objects, which, to some extent, proves the generalization ability of our method.

Simultaneously, the runtime statistics are shown at the bottom of Figure 5. Direct reconstruction methods like LCT require no iteration and, thus, shows fastest computation. DO-NLOS improves quality but demands hundreds of seconds due to heavy iterative optimization. In contrast, MD-NLOS demonstrates high computational efficiency, approaching the level of LCT, completing reconstructions in only approximately 1 s, while ensuring high reconstruction quality.

Furthermore, compared with our experimental data, due to the decreased spatial dimensionality of the data from 128 × 128 to 64 × 64 and 32 × 32, the time consumed by MD-NLOS reached the level of direct methods. This demonstrates that our method is premised on a strategic compromise: reducing image pixel points to attain computational efficiency rivaling direct methods, when this less pixel point is adequate for fundamental information. More importantly, it allows for substantial acquisition time savings through undersampling, while still faithfully reconstructing both image clarity and spatial details.

For simplicity, we consider a discretization in which the hidden scene volume is represented on an *N*×*N*×*N* grid, and the relay wall is sampled at *N*×*N* spatial locations. The measurement at each location is a temporal histogram with *N* bins. In the LCT reconstruction, it first applies a reparameterization along the time dimension (*O*(*N*^3^)). The resulting volume is then processed by a 3D Fast Fourier Transform (FFT) (*O*(*N*^3^*log*⁡*N*)), multiplied element-wise by a frequency-domain kernel (*O*(*N*^3^)), and transformed back via an inverse 3D FFT (*O*(*N*^3^*log*⁡*N*)), followed by a second time-domain transformation (*O*(*N*^3^)). Therefore, the end-to-end complexity is *O*(*N*^3^*log*⁡*N*). In the LCT-based MD-NLOS solver, each iteration consists of three main components: one application of the forward operator *A*, one application of its adjoint *A*^⊤^, and one projection step. Both *A* and *A*^⊤^ are implemented through the LCT pipeline. The projection step corresponds to an element-wise soft-thresholding operation, which requires a single pass over the 3D volume and thus costs *O*(*N*^3^). Consequently, the per-iteration complexity is *O*(*N*^3^*log*⁡*N*). Compared with direct LCT reconstruction, MD-NLOS incurs no additional computational overhead. The complexity comparison between other algorithms and our algorithm is shown in Table 1.

**Table 1:** Computational complexity comparison of confocal NLOS imaging algorithms

Method	FK	LCT	FBP	DO-NLOS	Ours
Complexity per iteration	O(N^3^ log N)	O(N^3^ log N)	O(N^5^)	O(N^3^ log N)	O(N^3^ log N)

### Resolution limits

We experimentally investigated the resolution limit of the proposed method. For confocal NLOS imaging system, the lateral resolution Δ*x* can be expressed as^4^:

(Equation 12) $$\begin{array}{c} \Delta x=\frac{c\gamma \sqrt{w^{2}+z^{2}}}{2w} \end{array}$$

where *c* is the speed of light, *γ* is the full width at half maximum (FWHM) of the temporal jitter of the detection system, *w* is the half-width of the scanning area, and *z* is the distance between the hidden object and the wall. Calibrated by Gaussian function, *γ* is 80.016 ps (see Figure S3), *w* is 0.8 m, and *z* is 0.5 m; thus Δ*x* is ∼1.92 cm. A dense scan of 128 × 128 sampling points over an 80 × 80 cm^2^ wall area was used as the full-sampling reference. According to the Nyquist sampling theorem, the sampling interval Δ*d* should satisfy:

(Equation 13) $$\begin{array}{c} \Delta d<\frac{\Delta x}{2} \end{array}$$

**Figure 6:**
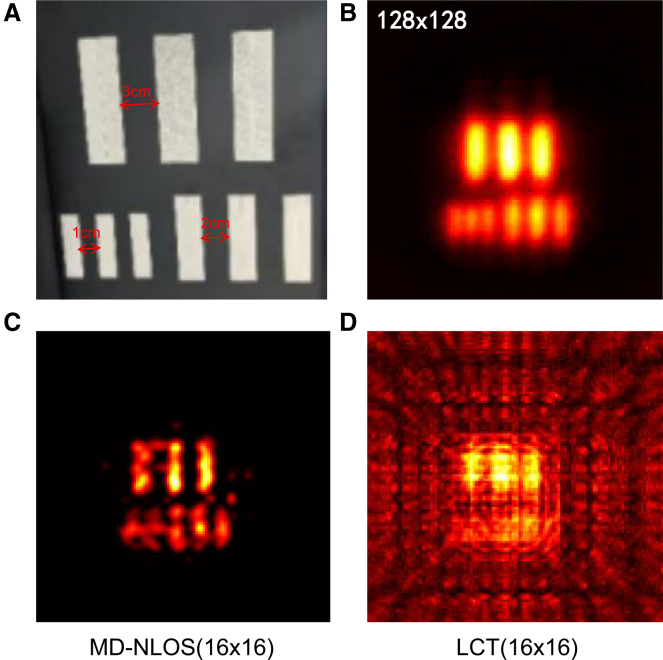
Comparison of LCT and the proposed method on an experimental resolution target (A) Object. (B) 128 × 128 full sampling with LCT. (C) 16 × 16 undersampling with MD-NLOS. (D) 16 × 16 undersampling with LCT.

In this configuration, the sampling interval is 0.625 cm, which is sufficiently smaller than 0.96 cm and, therefore, meets the full-sampling requirements. For resolution evaluation, a resolution target consisting of three groups of line patterns with separations of 3, 2, and 1 cm was employed. The reconstruction results are shown in Figure 6. It can be observed that, under 16 × 16 sampling, our method consistently outperformed LCT and was still able to clearly resolve line structures with a 2 cm separation. This result demonstrates not only the effectiveness of sparse recovery but also the achieved reconstruction performance in MD-NLOS, approaching the theoretical resolution limit of the system.

## Discussion

In summary, the proposed MD-NLOS imaging method provides a physically grounded yet computationally efficient approach to facilitate real-time hidden-scene reconstruction. Within the combined LASSO and spectral-filtering framework, even the simplest form of gradient descent is sufficient to achieve fast, stable, and noise-robust convergence. Furthermore, the simplicity of the algorithm makes it highly compatible with parallel computation on GPUs. Another key advantage of the framework is its generality. Although developed in the context of transient-based NLOS imaging, the same model decomposition strategy can be extended to other inverse imaging problems (for example, computed tomography^51^). Our approach is particularly well suited for scenarios that demand real-time performance, where physical models are well defined but the available data are limited or incomplete. The balance our method achieves in acquisition speed, computational efficiency, and reconstruction quality holds considerable promise for future NLOS imaging systems. For instance, when combined with front-end techniques like time-stretching,^52^^,^^53^ our approach could bring the real-time capability of NLOS imaging significantly closer to that of conventional line-of-sight imaging.

### Limitations of the study

Although the proposed method achieved a reasonable trade-off among computational efficiency, acquisition efficiency, and reconstruction resolution, its performance remains dependent on target complexity. Specifically, for structurally simple or highly sparse targets, the proposed framework can maintain satisfactory reconstruction quality under a limited sampling budget. However, for more complex objects with richer geometric and textural details, the combination of undersampling and sparsity-driven regularization may lead to the loss of high-frequency information, resulting in blurred or oversmoothed fine details. Future work will, therefore, focus on improving real-time performance for complex targets, for example by incorporating stronger yet lightweight priors (e.g., structure-aware or content-adaptive regularization) while preserving the computational constraints required for real-time reconstruction. Moreover, because the hidden-scene information is inferred from highly complex indirect (multipath) measurements, the reconstructions may still exhibit artifacts or spurious structures under certain conditions. Mitigating these artifact-related effects is an important direction for future investigation and improvement.

## Resource availability

### Lead contact

Requests for further information and resources should be directed to and will be fulfilled by the lead contact, Xiangang Luo (lxg@ioe.ac.cn).

### Materials availability

This study did not generate new unique reagents.

### Data and code availability

• The data and code that support the findings of this study are available from the corresponding author upon reasonable request.
• The data reported in this paper will be shared by the lead contact upon reasonable request.
• This paper does not report original code.
• Any additional information required to reanalyze the data reported in this paper is available from the lead contact upon request.

## Acknowledgments

The authors want to thank the State Key Laboratory of Optical Field Manipulation Science and Technology from 10.13039/501100011332University of Chinese Academy of Sciences to realize the experimental work. This work was supported by the 10.13039/501100001809National Natural Science Foundation of China (U24A6010, and 52488301) and the 10.13039/501100018542Natural Science Foundation of Sichuan (2023NSFSC0029).

## Author contributions

Conceptualization, P.Y. and Z.W.; methodology, P.Y.; investigation, P.Y. and Z.W.; writing – original draft, P.Y.; writing – review & editing, P.Y. and Z.W.; funding acquisition, Y.G.; resources, Y.G., X.L., M.P., H.G., M.X., F.Z., and X.L.; supervision, Y.G., M.P., and X.L.

## Declaration of interests

The authors declare no competing interests.

## Declaration of generative AI and AI-assisted technologies in the writing process

During the preparation of this work, the authors used ChatGPT in order to make the language smooth. After using this tool or service, the authors reviewed and edited the content as needed and take full responsibility for the content of the publication.

## STAR★Methods

### Key resources table

**Table:** 

REAGENT or RESOURCE	SOURCE	IDENTIFIER
**Deposited data**		

“Bunny”、 “SU” 、“Dot”、 “S”	N/A	Mendeley Data:https://doi.org/10.1038/nature25489
“dragon”、“bike”	N/A	Mendeley Data:https://doi.org/10.1145/3306346.3322937

**Software and algorithms**		

LCT	N/A	Mendeley Data:https://doi.org/10.1038/nature25489
FBP	N/A	Mendeley Data:https://doi.org/10.1038/ncomms1747
DO-NLOS	N/A	Mendeley Data:https://github.com/Miaomiaoliyyygj/DONLOS.
FK	N/A	Mendeley Data:https://doi.org/10.1145/3306346.3322937

**Other**		

PC	NVIDIA GeForce RTX 4060 graphics processor	N/A
SPAD	PDM	N/A
TCSPC	PicoHarp 300	N/A
Mechanical galvanometer	Thorlabs’ GVS002	N/A

### Experimental model and study participant details

The experimental model is built upon the LCT inversion framework for NLOS imaging. Specifically, the LCT-based forward model is reformulated as a LASSO optimization problem, which solves for the hidden scene by minimizing a data fidelity term regularized by an ℓ₁-norm penalty to promote sparsity. All reconstructions are obtained via numerical simulations using this optimization scheme. Therefore, no study participant details are applicable.

### Method details

#### Details on the LASSO framework

##### Derivation of the optimization function

Under the Gaussian assumption, we can write our observation model as:

(Equation 14) $$\begin{array}{c} y=Af+\varepsilon ,\varepsilon ∼N\left(0,\sigma^{2}I\right) \end{array}$$

Under the Gaussian model (14), the probability of observing a particular vector of counts *y* is given by:

(Equation 15) $$\begin{array}{c} p\left(y_{i}|Af\right)=\frac{1}{\sqrt{2\pi \sigma^{2}}}\exp\left(-\frac{{\left(y_{i}-e_{i}^{T}Af\right)}^{2}}{2\sigma^{2}}\right) \end{array}$$

The negative log likelihood function is:

(Equation 16) $$\begin{array}{c} F\left(f\right)=\frac{1}{2\sigma^{2}}\sum_{i=1}^{m}{\left(y_{i}-e_{i}^{T}Af\right)}^{2} \end{array}$$

##### Physical interpretation of the denoising process

Regarding the denoising process, the *L*_1_ norm regularization term in LASSO causes the reflectance *ρ* at a specific location to be compressed to zero if its contribution to reducing the reconstruction error (i.e., the fidelity term) is insignificant. This effectively treats its contribution as mere noise, thereby achieving feature selection and promoting a sparse solution. This process is determined by the correlation of the residual at that location. For ease of understanding, we write the equation in the form before decoupling:

(Equation 17) $$\begin{array}{c} \partial f\left(\rho \right)=A^{T}\left(A\rho^{k}-\tau \right)+\alpha \text{sgn}\left(\rho_{i}\right)\;\forall \;i \end{array}$$

where: $f\left(\rho \right)=\frac{1}{2}|\left|A\rho -\tau \right|{|}_{2}^{2}+\alpha {\left\|\rho \right\|}_{1}$, $\text{sgn}\left(\rho_{i}\right)=\left\{ \begin{array}{c} 1,\rho_{i}>0\\ \left[-1,1\right],\rho_{i}=0\\ -1,\rho_{i}<0 \end{array}\right.$. The term *Aρ*^*k*^-*τ* is the residual, indicating whether the current solution *ρ*^*k*^ has fully exploited the information in the dictionary *A*. If fully exploited, the inner product with the corresponding column in (*Aρ*^*k*^-*τ*) becomes small, which means that noise dominates now and the reflectance *ρ*_*i*_ at that location can be compressed to zero; conversely, if not fully exploited, the inner product value is large, and the location is retained. The threshold for judging the magnitude of this inner product is controlled by *α*; if the absolute value is greater than α, it is retained, otherwise set to zero. Through this mechanism, LASSO regularization automatically suppresses noise and highlights the positions of the hidden object during reconstruction, making the physical interpretation of the solution clearer. In this paper, the comparison is between $\left|z_{i}^{k}\right|$ and *α*/*λ*.

##### Decoupling the linear relationship and the origin of the quadratic term from the second-order term in Taylor expansion

To decouple the relationship between *ρ* and *τ* in Equation 5, let $g\left(\rho \right)=\frac{1}{2}\|A\rho -\tau {\|}_{2}^{2}$ and perform a first-order Taylor expansion around *ρ*^*k*^:

(Equation 18) $$\begin{array}{c} g\left(\rho \right)\approx g\left(\rho^{k}\right)+\nabla g{\left(\rho^{k}\right)}^{T}\left(\rho -\rho^{k}\right) \end{array}$$

Then Equation 5 can be rewritten as:

(Equation 19) $$\begin{array}{c} \underset{\rho }{\arg\min}\;\alpha {\left\|\rho \right\|}_{1}+g\left(\rho^{k}\right)+\nabla g{\left(\rho^{k}\right)}^{T}\left(\rho -\rho^{k}\right)+\lambda {\left\|\rho -\rho^{k}\right\|}_{2}^{2},s.t.\;\rho \geq 0 \end{array}$$

Since *g*(*ρ*^*k*^) is a constant term, let $g\left(\rho^{k}\right)=\lambda \|\nabla g(\rho^{k}{)\|}_{2}^{2}$ to facilitate completing the square.

Then the optimization Equation 5 can be written as:

(Equation 20) $$\begin{array}{c} \underset{\rho }{\arg\min}\;\alpha {\left\|\rho \right\|}_{1}+\lambda \|\rho -\rho^{k}+\frac{1}{2\lambda }\nabla g\left(\rho^{k}\right){\|}_{2}^{2},s.t.\;\rho \geq 0 \end{array}$$

Actually, if we perform a second-order Taylor expansion of *g*(*ρ*), the last term introduced is precisely the proximal term $\frac{\lambda }{2}{\left\|\rho -\rho^{k}\right\|}_{2}^{2}$. And *λ* corresponds to its second derivative, making the introduction of this term very natural.

We assume that the sought *ρ*^*k*+1^ is known. This is essentially no different from knowing *ρ*^*k*^ and finding *ρ*^*k*+1^. According to the definition of gradient flow:

(Equation 21) $$\begin{array}{c} \frac{d}{dt}\rho \left(t\right)=\frac{\rho \left(t+h\right)-\rho \left(t\right)}{h}=\frac{\rho \left(t\right)-\rho \left(t-h\right)}{h}=-\nabla g\left(\rho \left(t\right)\right) \end{array}$$

From this, we can derive:

(Equation 22) $$\begin{array}{c} {\rho^{k+1}=\rho^{k}-h\nabla g\left(\rho^{k}\right)⟺\rho }^{k+1}=\rho^{k}-h\nabla g\left(\rho^{k+1}\right) \end{array}$$

Therefore, this assumption merely uses a different difference quotient method. It is essentially identical to the gradient descent method.

##### The selection of NLOS models

Classical NLOS reconstruction algorithms can be broadly categorized into two classes: those based on geometric constraints and those grounded in wave optics.

Filtered back-projection (FBP) belongs to the former category. It operates by back-projecting each time-of-flight histogram acquired at a scanning point along the corresponding ellipsoidal surface into the reconstruction volume. A single measurement contributes responses to all voxels intersected by that specific ellipsoid. As the illumination and detection positions scan across the relay wall, the ellipsoids from multiple measurements continuously intersect in the vicinity of the true target locations. Consequently, voxels corresponding to actual scatterers accumulate higher responses, progressively revealing the spatial contours of the hidden object. The imaging process can be formally expressed as:

(Equation 23) $$\begin{array}{c} I_{\text{fin}}=I_{R}*psf\\ psf=\overset{¯}{K}*K \end{array}$$

where *I*_*R*_ represents the spatiotemporal measurement on the relay wall, *K* denotes the back-propagation kernel, $\overset{ˉ}{K}$ its conjugate counterpart, psf the system point spread function, and ∗ the convolution operator. Crucially, *K* represents a voxel-dependent space-variant kernel, rather than a spatially invariant convolution kernel. This implies that the back-projection kernel in FBP lacks shift-invariance, preventing the pre-computation and reuse of a fixed kernel—a technique employed by algorithms such as LCT through variable substitutions. Consequently, FBP incurs significant computational cost. Furthermore, FBP yields a probabilistic distribution of the object rather than an exact numerical solution, often resulting in suboptimal reconstruction quality. In contrast, LCT achieves discretization via resampling operators and provides a closed-form solution.

Frequency-domain methods based on wave optics, such as the FK migration algorithm, offer higher physical fidelity. The core step in FK methods involves a Stolt interpolation operation that transforms transient measurement data into a three-dimensional volumetric representation of the hidden object. However, when formulating the reconstruction as an iterative optimization problem, computing the gradient necessitates the adjoint of the Stolt interpolation operator. In the FK framework, the adjoint of the forward operator cannot be explicitly defined. By comparison, the forward operator in LCT is independently characterized by a point spread function, making its adjoint operator more straightforward to define and facilitating integration with structural priors such as sparsity.

Finally, it is important to recognize that undersampled NLOS imaging is inherently an ill-posed inverse problem. The challenges stem from the high dimensionality of the reconstruction space, sensitivity to noise, information loss due to sparse sampling, and the lack of sufficient structural priors within the measurement operator. LCT, by employing a confocal experimental configuration, effectively reduces the dimensionality from the 5D transient data in FBP to a 3D volume. Regularization techniques, such as $l_{1}$-norm penalties, are incorporated to enhance noise robustness and promote sparse recovery.

##### Principles of NLOS imaging based on geometric constraints

As illustrated in Figure S1A, A pulsed laser beam is directed onto a scanning position on a visible relay wall. Following diffuse reflection at the wall, the light propagates into the occluded region, indirectly illuminating the hidden target. The target subsequently scatters the incident light, generating echo signals that return to the relay wall, where their arrival times are recorded by a single-photon detector. Given the one-to-one correspondence between the time-of-flight of light and the geometric path length, the temporal information embedded in the echoes can be exploited to establish geometric constraints on the hidden scene, thereby enabling the reconstruction of the target’s position and shape.

More specifically, when the hidden target possesses a finite volume, it can be discretized into a collection of scattering points. Each such point behaves as a secondary source. Owing to variations in their distances to the detector, the photons originating from different points arrive at distinct times. Consequently, for a given pixel on the relay wall, the detector records multiple echo components arriving at different instants, as depicted in Figures S1B and S1C. This indicates that the transient data acquired at a single pixel represent the temporal superposition of responses from numerous discrete scattering points on the hidden target.

To elucidate the relationship between time-of-flight information and the spatial location of a hidden target, we first consider the determination of the three-dimensional position of an isolated scattering point. In the non-confocal configuration shown in Figure S1D, the laser illumination point and the detection point on the relay wall are spatially separated and denoted as *w*_1_ and *w*_2_, respectively, with *w*_1_≠*w*_2_. Let the source position be *l* and the detector position be *v*. For an arbitrary discrete scattering point *o* on the hidden target, when the system registers an echo signal within a specific time bin, the total optical path length *L* traversed by the light is given by:

(Equation 24) $$\begin{array}{c} L=\left\|lw_{1}\left\|+\right\|w_{1}o\left\|+\right\|ow_{2}\left\|+\right\|w_{2}v\right\| \end{array}$$

This path length satisfies the time-of-flight relation:

(Equation 25) $$\begin{array}{c} L=ct \end{array}$$

where *c* denotes the speed of light. As the positions *l*, *v*, *w*_1_, and *w*_2_ are known *a priori*, the distances ‖*lw*_1_‖ and ‖*w*_2_*v*‖ are constants. Subtracting these fixed path lengths from the total yields the effective optical path length associated with the hidden target:

(Equation 26) $$\begin{array}{c} d=\left\|w_{1}o\right\|+\left\|ow_{2}\right\|=ct-\left\|lw_{1}\left\|-\right\|w_{2}v\right\| \end{array}$$

From a single echo measurement, it follows that the possible locations of the target point *o* lie on an ellipsoid with foci at *w*_1_ and *w*_2_ and a major axis length of *d*.

A single time-of-flight measurement thus constrains the target point to this ellipsoidal surface but is insufficient to uniquely determine its three-dimensional coordinates. By fixing the detection point and varying the illumination point, additional ellipsoidal constraints corresponding to different focal pairs can be generated for the same hidden point, thereby enabling its unambiguous spatial localization. Extending this concept to a hidden target composed of numerous discrete scattering points, the time-of-flight information recorded at each pixel on the relay wall corresponds to a set of ellipsoidal constraints associated with these points. By scanning multiple positions on the relay wall and jointly utilizing the time-of-flight data from all pixels, the spatial distribution of the scattering points can be progressively recovered, achieving tomographic reconstruction of the entire hidden target. It is noteworthy that in a confocal configuration, the geometric constraint reduces to a spherical surface.

##### Calibration of the total time jitter

The transient histograms are acquired using time-correlated single-photon counting (TCSPC) with a bin width of 4 ps. To estimate the full width at half maximum (FWHM) of the instrument temporal response, we aggregate every eight bins into one unit and then fit the resulting temporal profile with a Gaussian function, as shown in Figure S4. The measured overall timing jitter of the system is 80.016 ps, which includes contributions from the SPAD detector temporal resolution (approximately 70 ps) and the laser pulse width (approximately 20 ps). Following Wu et al.,^4^ the additional broadening induced by the field of view (FOV) can be estimated by subtracting the above independent components in quadrature:

(Equation 27) $$\begin{array}{c} \sigma_{\text{FOV}}=\sqrt{80^{2}-70^{2}-20^{2}}ps\approx 33ps \end{array}$$

The above three parts are the main components of overall time jitter.

##### Details on the parallel computing technique

the MD-NLOS method was implemented in parallel using MATLAB’s gpuArray mechanism. Specifically, the transient data, system matrix, reflectivity function, and related intermediate variables were transferred to the GPU in a unified manner. During the iterative process, operations such as frequency-domain point-wise multiplication, non-negativity projection, and soft-thresholding were all performed directly on gpuArray data, with MATLAB automatically invoking the underlying GPU parallel computing resources for acceleration.

### Quantification and statistical analysis

Quantitative evaluation was performed using peak signal-to-noise ratio (PSNR), structural similarity index (SSIM), and root-mean-square error (RMSE), together with the reconstruction runtime. All analyses were conducted in MATLAB (MathWorks), and figures were assembled for presentation in Microsoft PowerPoint. Reconstruction runtime was measured by repeating each experiment three times under identical settings and reporting the average value across the three runs.

### Additional resources

This study did not generate additional online resources.

## Supplemental information

Document S1. Figures S1–S4, Table S1, and supplemental references
