# Supplementary material for: A model decomposition method for the real-time non-line-of-sight imaging
Source: iScience. 2026 Apr 21;29(6):115828. doi: 10.1016/j.isci.2026.115828 (PMC13156713; doi:10.1016/j.isci.2026.115828)
Supplement: Document S1. Figures S1–S4, Table S1, and supplemental references [file mmc1.pdf]

iScience, Volume *29*

Supplemental information

A model decomposition method for the real-time non-line-of-sight imaging

Peng Yang, Zewei Wang, Yinghui Guo, Xiaoying Li, Mingbo Pu, Hengshuo Guo, Mingfeng Xu, Fei Zhang, Yuanmao Wang, and Xiangang Luo

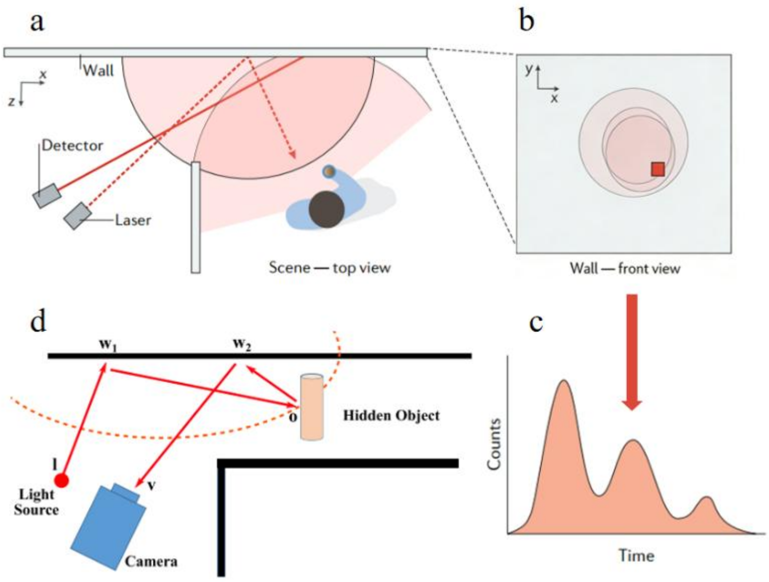

Figure S1. Principles of Time-of-Flight Based Single-Photon Non-Line-of-Sight Imaging[1-2], related to Figure 1. a) Scattering mechanism. b) Schematic of the waves scattered by the hidden object at the relay wall. c) Schematic time trace of the photon counts observed at a given pixel on the relay wall. The peaks correspond to scattering spherical waves that expand over time. d) Non-confocal experimental configuration.

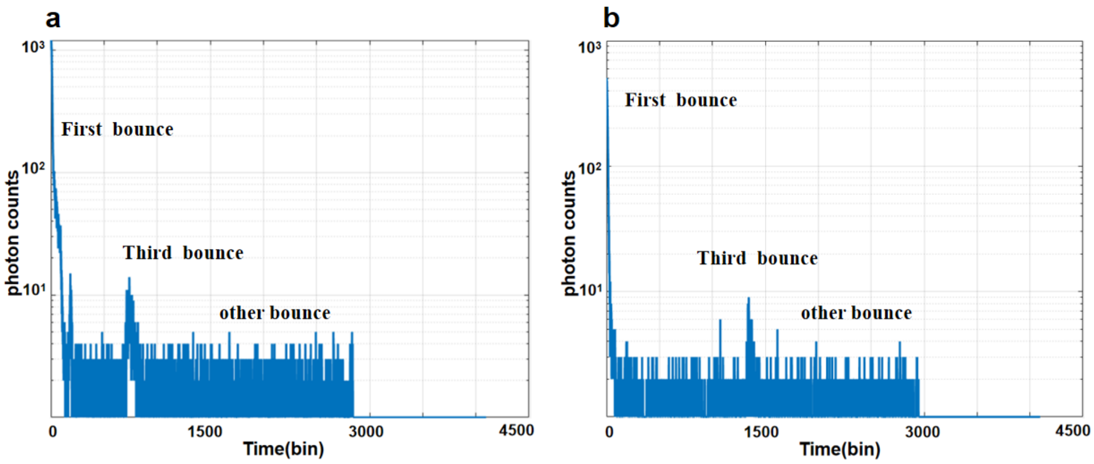

Figure S2. Signal of bounced photons, related to Figure 2. a) Photon counts recorded from the experimental object “E”. b) Photon counts recorded from the experimental object “I”. The hidden scene is shown in Figure 3a, where two letters, “I” and “E”, are placed in a corner. The letter “I” faces the first relay surface at a distance of 0.5 m, whereas the letter “E” faces the second relay surface at a distance of 0.8 m. The first relay surface is a smooth white board, and the second relay surface is a rough white-painted wall. According to O’Toole et al.[3], photon counts of diffusely reflected light from the relay surface decay approximately with the inverse square of distance, leading to severe attenuation during propagation. As a result, the photon counts associated with the first and third reflections already differ by about two orders of magnitude, whereas signals from subsequent higher-order reflections are nearly indistinguishable from noise.

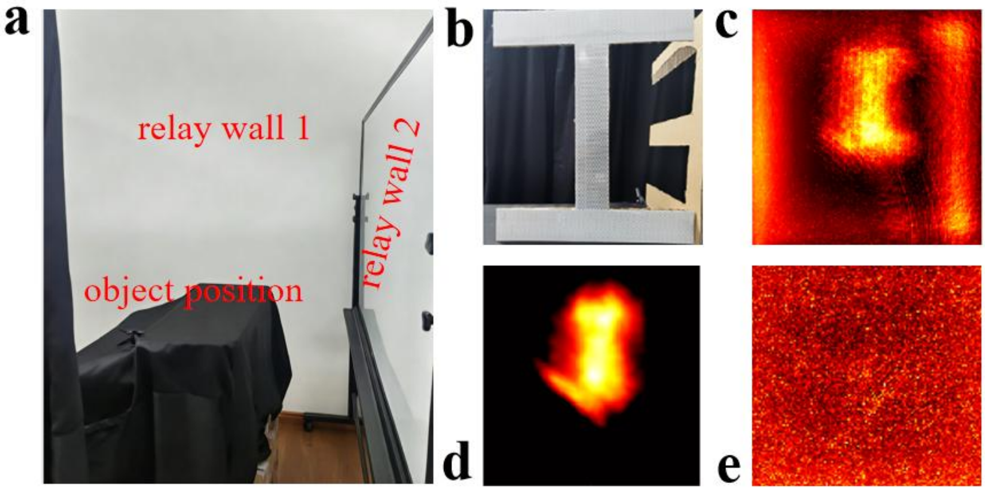

Figure S3. Multiple reflection experiment, related to Figure 2. a) Experimental layout of the multiple-reflection setup, where two relay walls introduce occlusion and complex multipath light transport. In this configuration, photons undergo at least five reflections before returning to the detection system. b) Reference appearance of the experimental target “I”. c) Reconstruction obtained by LCT under full 128×128 sampling, which recovers the dominant target structure but still exhibits noticeable background noise and artifacts, indicating the increased difficulty of reconstruction in multiple-reflection scenes. d) Reconstruction obtained by MD-NLOS under 24×24 undersampling. e) Reconstruction obtained by LCT under the 24×24 undersampling condition. Under severe undersampling, the conventional LCT result is dominated by noise and speckle-like artifacts, making the target structure hardly identifiable. By contrast, MD-NLOS preserves the main energy distribution and the dominant vertical stroke of the letter “I”, demonstrating improved robustness to undersampling in the presence of complex multipath. Nevertheless, the thin horizontal strokes are substantially attenuated or missing, suggesting a loss of high-spatial-frequency details.

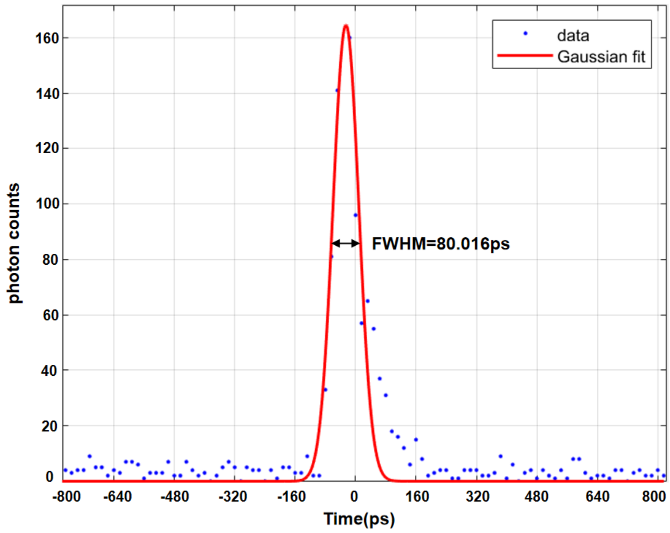

Figure S4. Stacked data measured by TCSPC, related to Figure 6.

	Method	PSNR	RMSE	SSIM
**Using 256 points**	LCT	11.4802	0.2667	0.4731
	FBP	10.2745	0.3064	0.2173
	FK	15.3955	0.1699	0.6209
	Ours	12.8344	0.2282	0.7613
**Using 64 points**	LCT	9.2338	0.3454	0.1273
	FBP	9.6408	0.3296	0.0726
	FK	13.0759	0.2219	0.2842
	Ours	12.6549	0.2329	0.7564
**Using 36 points**	LCT	8.8745	0.3600	0.0382
	FBP	8.0895	0.3940	0.0039
	FK	11.6855	0.2605	0.1342
	Ours	11.9328	0.2531	0.7352

Table S1. Statistical data of PSNR, RMSE, and SSIM under undersampling rates using different methods, related to Figure 4.

REFERENCES

1. Maeda, T., Satat, G., Swedish, T., Sinha, L., and Raskar, R. (2019). Recent advances in imaging around corners. arXiv 1910.05613. 10.48550/arXiv.1910.05613.

2. Faccio, D., Velten, A., and Wetzstein, G. (2020). Non-line-of-sight imaging. Nat. Rev. Phys. 2, 318–327. 10.1038/s42254-020-0174-8.

3. O'Toole, M., Lindell, D.B., and Wetzstein, G. (2018). Confocal non-line-of-sight imaging based on the light-cone transform. Nature 555, 338–341. 10.1038/nature25489.
